# Barriers and facilitators of parent−adolescent communication on sexual health and relationships among the UK population: A study protocol

**DOI:** 10.1002/hsr2.1975

**Published:** 2024-03-13

**Authors:** Laura O. Joseph, Pascal L. Navelle, Chinwendu C. Ngozi, Dorothy Hannis, Rebekah McNaughton, Lawrence A. Nnyanzi

**Affiliations:** ^1^ School of Health and Life Sciences Teesside University Middlesbrough UK

**Keywords:** barriers, facilitators, parent–adolescent communication, sexual reproductive health, UK

## Abstract

**Background and Aims:**

Improving the sexual and reproductive health of adolescents remains a public health priority. Effective communication between parents and adolescents regarding sexual health and relationships is important and could influence adolescents to adopt safer sexual behaviors. However, several barriers can impede this communication in the UK population. The aim of the review is to explore barriers and facilitators, as well as wider determinants of parent–adolescent communication (PAC) on sexual health and relationships. The review will explore possible interventions aimed at promoting PAC on sexual health and relationships.

**Methods:**

The review will focus on exploring barriers and facilitators of PAC on sexual health and relationships in the United Kingdom. This study will synthesize qualitative literature to understand the significance of parent–adolescent sexual and reproductive health communication, the challenges and facilitators to effective communication in the United Kingdom. Using a detailed predetermined search strategy, the study will search for relevant studies from CINAHL, MEDLINE, PsycINFO, EMBASE, SCOPUS, Web of Science, and gray literature on Google Scholar. The Critical Appraisal Skills Program checklist will appraise the included studies' methodological quality. A thematic synthesis approach will be used to synthesize data from included studies.

**Conclusion:**

Findings from the systematic review are expected to give information on the barriers and facilitators of PAC on sexual health and relationships which will further optimize interventions to improve this type of communication and guide future research in understanding this area.

**Systematic review registration:**

The review has been registered with the International Prospective Register of Systematic Reviews CRD (PROSPERO) (CRD42022351697).

## 1 BACKGROUND

Adolescent Sexual and Reproductive Health Education is an essential concern to the public health sector as over one‐sixth of the world's population are adolescents.^1^ The World Health Organization (WHO)^2^ has defined adolescents as individuals aged 10–19 years. Nearly half of the world's total population are young people under 25 years of age.^3^ Adolescence is a time characterized by physical, emotional, and psychological changes that can cause young people to be vulnerable to health and social problems.^4^ Equally, according to the WHO, sexual health is not just the absence of disease, malfunction, or infirmity but also a condition of physical, emotional, mental, and social well‐being in relation to sexuality.^2^

The parent–adolescent dyad about communication on relationships and sexual health has become a contemporary public health issue as studies have shown it is effective in setting a solid foundation for an adolescent to have good sexual health and sexual behavior.^4^, ^5^ Over the last few years, sexual activity around the globe has been on a steady rise among the adolescent population,^6^, ^7^ which makes Adolescent Sexual Reproductive Health (ASRH) a global Public Health issue.

Approaches to promote healthy adolescent sexual behavior in the United Kingdom includes sexual education, policy frameworks, over‐the‐counter contraceptives, sexual health clinics, and access to sex and sexuality information.^4^, ^8^ Although there is a generalization that adolescents in the United Kingdom have access to sex and sexuality information,^8^ the extent to which the knowledge (sexual risks) translates into practicality (safe sex practices) is not evident.^8^, ^9^, ^10^, ^11^, ^12^

Findings from the literature point to how family systems serve as the central agent of socialization for children.^4^, ^12^ The premise on which the aforementioned occur is that parents are in regular proximity and contact with their children, thus can shape their behavior, provide guidance, and influence social contexts in which they develop.^4^ In their study, Downing et al.^13^ further add that parent–adolescent communication (PAC) timing is critical, highlighting that communication that occurs before adolescents begin engaging in sexual activities is more effective.

Poor PAC has been identified to have numerous potential risk factors.^14^, ^15^ One is parental awareness, while others are cultural concerns like parents feeling embarrassed.^4^ To decide when, how, or on what occasions parents should commence the discussions, ongoing research projects demonstrating cutting‐edge PAC methods are required.^16^, ^17^, ^18^ As a result, most teenage sexual issues are typically misdirected by their sex‐specific peer groups.^19^ The topic of conversation,^20^ parents' self‐efficacy for communication,^21^ parent–adolescent relationship,^22^ communication patterns,^23^ media,^24^, ^25^ and peer group factors are just a few of the factors that have been shown in prior research to influence PAC about sexuality.

Results from research suggest that parents in high‐resource settings, including Europe and America, are comfortable and have open conversations about sex and Sexual Reproductive Health (SRH) issues with their adolescent children.^26^ Furthermore, they elucidate that Western countries believe that indulging in PAC can significantly increase the chances of adolescents engaging in safer sex behavior, including delaying sex and increasing use of condoms and contraceptives.^26^, ^27^, ^28^, ^29^ However, findings from existing studies on PAC in the United Kingdom have suggested otherwise.^20^

A common theme discovered across the UK is that discussions between parents and adolescents on SRH issues occur but not often and are characterized by parents feeling unprepared and unable to address the sensitive matters around sex, sexuality, and other SRH issues.^20^ Also, recent studies around PAC in the United Kingdom have focused on digital sexual health interventions,^30^ with limited evidence existing about the barriers and facilitators of PAC in the United Kingdom. This systematic review aims to highlight barriers and facilitators to PAC, particularly in the United Kingdom. Additionally, the review will explore the wider determinant factors influencing this communication.

## 2 METHODS

### 2.1 Protocol and registration

The review protocol has been carried out under the guidelines of the preferred reporting items for the Systematic Reviews and Meta‐Analyses Protocols (PRISMA‐P) checklist.^31^, ^32^ The review has been registered in the International Prospective Register of Systematic Reviews (PROSPERO) CRD42022351697. The protocol states the objectives and methodology to be conducted and focuses on exploring the barriers and facilitates influencing PAC on sexual health and relationship. Additionally, this review will examine the wider determinant factors influencing this communication.

### 2.2 Search strategy

A comprehensive electronic literature search will be carried out to explore the barriers and facilitators influencing PAC on sexual health and relationship. Although there are other databases on this topic, the following electronic databases will be searched: CINAHL, MEDLINE, PsycINFO, EMBASE, SCOPUS, Web of Science, and Google Scholar. These databases will be investigated using a comprehensive search approach/keyword. The selected databases are one of the largest databases that holds robust information pertaining to the proposed topic of interest. The rationale for searching these databases is to ensure access to specialized, quality‐controlled literature spanning various disciplines offering advanced search options, full‐text access, citation tracking, alert for efficient, and thorough literature reviews. Gray literature (non‐published, internal, or unreviewed articles, repositories) will also be searched. The reference lists of selected systematic reviews will be examined to determine whether they contain relevant papers that could be included in the review. Two researchers (L. J. and P. N.) will search the databases independently. The search terms that will be used in the search are related to “parents,” “adolescents,” “communication,” “sexual health,” “barriers,” “facilitators,” and the United Kingdom.” These keywords will then be combined using Boolean operators “AND” and “OR.” The Medical Subject Headings (MeSH) will also be utilized when searching various databases. The researchers will use the truncation signal (asterisk *) to search for all term alternatives. Supporting Information S1: Appendix Table 2 shows the preliminary search technique.

### 2.3 Eligibility criteria

The studies involving parents of UK adolescents who have had sexual health communication with their adolescents will be included in this review. The United Kingdom is a suitable choice for studying barriers and facilitators of PAC on sexual health and relationships among the population for several reasons. First, the UK has a diverse population, allowing a broad range of perspectives to be studied. Additionally, the UK has a well‐established National Health Service, which provides sexual health services and resources and a strong emphasis on sex education in schools. Furthermore, there is a wealth of existing research on sexual health and relationships in the United Kingdom, providing a foundation for further study. Additionally, the UK has a history of social and cultural changes regarding sexual health and relationships, which allows for exploring the impact of these changes on PAC. Thus, the studies that will be included in this review will define PAC, and studies that have identified common barriers and facilitators of PAC on sexual health and relationship. Studies published between 2015 and 2023 will be included as the field of ASRH has emerged over the last few years. Studies eligible will only be in English as authors are proficient in the language. This review excludes commentaries, editorials, symposium proceedings, and systematic reviews. Supporting Information S1: Appendix Table 1 demonstrates the inclusion and exclusion criteria using the population, interest, and outcomes (PIO) framework.

### 2.4 Selection of studies and screening

The records exported from all electronic databases will be managed using a systematic review management software Covidence.^33^, ^34^ The initial stage of the selection process will involve screening the title of retrieved articles based on the predetermined inclusion and exclusion criteria following the removal of duplicates.

**Figure 1:**
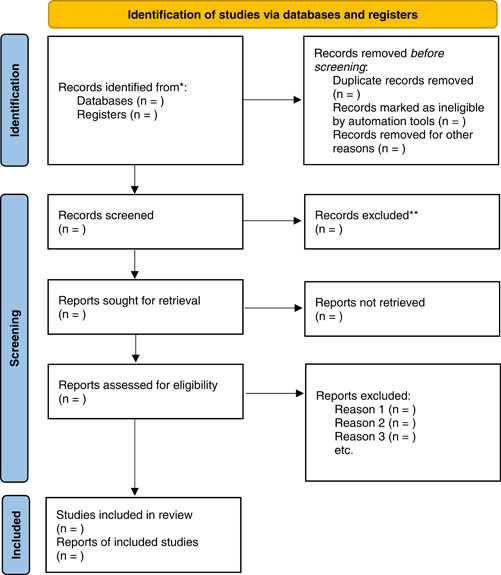
PRISMA database search flowchart for studies. Adopted from Page et al.^35^

The selected studies will subsequently be screened based on their abstracts at the second stage. Finally, full text of selected studies will be retrieved and evaluated against the inclusion and exclusion criteria. A predefined screening criteria will be developed to ensure the consistency of screening articles across the two reviewers (L. J. and P. N.), and pilot testing will be undertaken according to the eligibility criteria. Following the examination of the studies to determine the papers' eligibility, both reviewers will define outcome measures. Each reviewer will provide substantial reasoning for excluding studies. In a consensus meeting, a third reviewer will address any disagreement between the two reviewers. The third reviewer will be consulted to determine whether the study fits the inclusion eligibility criteria. The PRISMA flowchart diagram (Figure 1)^35^ will be used to show the study selection process. The studies that will be determined to be included will move to the methodological quality appraisal stage.

### 2.5 Data extraction and management process

Two independent reviewers (L. J. and P. N.) will use the Joanna Briggs Institute Data Extraction Form for Review for Systematic Reviews and Research Syntheses to extract data for the eligible/included studies.^36^ The data extraction tables of both reviewers will be compared to ensure that the systematic review contains all significant findings. A third reviewer will be involved if discrepant information is detected during the data extraction procedure. Supporting Information S1: Appendix Table 3 illustrates the preliminary data extraction table. Before writing the protocol, preliminary work on the topic was conducted to understand the robustness of the of the extraction form required for the study. The preliminary data extraction form includes the article's title, author, publication date, country of study, date of extraction, reviewer's name, purpose/goal of the study, study type, study population, study outcomes—barriers, and facilitators of PAC and study limitations.

### 2.6 Quality assessment of and risk of bias

To evaluate the risk of bias in qualitative studies, the Critical Appraisal Skills Program qualitative checklist tool will be used.^37^ The checklist (Supporting Information S1: Appendix Table 4) contains a series of questions. It prompts to appraise whether 10 elements (aims, methodology, design, recruitment strategy, data collection, reflexivity, ethical issues, data analysis, findings, and value of the research) are clear, adequately described and appropriate. The tool is divided into two halves. The first section of the instrument describes the study's findings in sufficient detail to support a bias risk assessment. The second section of the instrument provides a risk rating accomplished by ascribing a “yes,” “no,” or “can't tell.”

A “yes” is the highest possible ranking for this instrument, indicating a higher quality of study. Based on an analysis of the collected data, each study will be graded as having a high, moderate, low, or minimal risk of bias. If there are insufficient details, the risk of bias will be categorized as “can't tell” or the authors of the respective study will be contacted for additional information. Two reviewers (L. J. and P. N.) will evaluate the risk of bias of the included studies separately. In the event that two reviewers disagree, a third reviewer will be consulted. An additional table will contain information regarding the risk of bias for each of the listed studies.

### 2.7 Data synthesis strategy

First, a narrative synopsis of the review's results will be provided. Following this, we will perform a descriptive analysis of all the final included studies to detail their characteristics, such as research title, authors, publication year, study objective, study methodologies, sampling technique, participant characteristics, and study outcomes. A thematic synthesis^38^ will be undertaken in which the final studies will be categorized according to themes. The structure will be modified in accordance with emerging new themes. The thematic synthesis will be carried out using Thomas and Harden's thematic synthesis method.^38^ This will be used to extract underlying meanings from qualitative data, create analytical themes, and draw conclusions across studies. After becoming familiar with the data in the included studies, an inductive line‐by‐line coding approach will be used in this study.^38^ Some studies may not directly address the review questions; hence, this method will favor the data extraction based purely on review questions.

There are three stages to the thematic synthesis process: (i) Relevant qualitative data will be coded line‐by‐line as free codes, which will be named according to their meaning and substance; (ii) free codes are then looked at to see how they relate to one another and are classified into descriptive categories; (iii) analytical themes for thematic synthesis will emerge based on the underlying meanings of the descriptive categories. The coding will be carried out by the reviewer L. J., which was approved by the review team after a discussion with one additional reviewer (P. L. N). Analyzing linkages and interconnectivity led to synthesizing analytical themes and their subthemes will be carried out by L. J. and P. L. N Later, the reviewers will document their interpretations of the findings to identify emergent trends. Finally, the reviewers will emphasize possible facilitators and barriers to PAC on sexual health and the wider determinant factors influencing this communication.

## 3 DISCUSSION

There is a need for a deeper understanding of the potential facilitators and inhibitors influencing PAC on sexual health and relationship in the United Kingdom. The protocol will lead to a systematic review that synthesizes evidence on these barriers, facilitators, and other wider determinant factors. The review will expand our knowledge of how parents can overcome these barriers, including stigmatization, discrimination, fear of refusal, lack of privacy, and confidentiality. The results of a systematic review will be made available to the public.

## AUTHOR CONTRIBUTIONS

**Laura O. Joseph**: Conceptualization; data curation; formal analysis; investigation; methodology; project administration; resources; software; supervision; validation; visualization; writing—original draft; writing—review and editing. **Pascal L. Navelle**: Data curation; formal analysis; investigation; methodology; software; validation; writing—review and editing. **Chinwendu C. Ngozi**: Project administration; validation; visualization; writing—review and editing. **Dorothy Hannis**: supervision. **Rebekah McNaughton**: Supervision; visualization; writing—review and editing. **Lawrence A. Nnyanzi**: Supervision; validation; visualization; writing—review and editing.

## CONFLICT OF INTEREST STATEMENT

The authors declare no conflict of interest.

## ETHICS STATEMENT

The researchers have not applied for any ethical approval, as no identifiable information will be accessed during the review. Also, data will be collected from an open research repository.

## TRANSPARENCY STATEMENT

The lead author Laura O. Joseph affirms that this manuscript is an honest, accurate, and transparent account of the study being reported; that no important aspects of the study have been omitted; and that any discrepancies from the study as planned (and, if relevant, registered) have been explained.

## Supporting information

Supporting information.

## Data Availability

Data sharing does not apply to this article because no data sets are generated.
