# Supplementary material for: Barriers and facilitators of parent−adolescent communication on sexual health and relationships among the UK population: A study protocol
Source: Health Sci Rep. 2024 Mar 13;7(3):e1975. doi: 10.1002/hsr2.1975 (PMC10933652; doi:10.1002/hsr2.1975)
Supplement: Supplementary file 1 — Supporting information. [file HSR2-7-e1975-s001.docx]

**Appendices**

**Table 1:** Inclusion and exclusion criteria

| Prompt | Inclusion criteria | Exclusion criteria |
| --- | --- | --- |
| Population | Parents of adolescents, mothers, fathers, caregivers, adoptive parents | All other parents, grandparents |
| Interest | Parent-adolescent communication on sexual health and relationships | Studies that include parent-adolescent communication but do not discuss barriers and facilitators |
| Context | Studies undertaken in the UK | Studies undertaken outside the UK |
| Outcomes | Barriers of PAC on sexual health and relationship.  Facilitators of PAC on sexual health and relationship | Outcomes not related to:  Barriers of PAC on sexual health and relationship.  Facilitators of PAC on sexual health and relationship |
| Type of study | Qualitative studies | Dissertations, commentaries, editorials, proceedings, systematic reviews and narrative studies |
| Country | United Kingdom | Outside United Kingdom |
| Time Period | Present date – 2015 | Studies published before 2015 |
| Language | Studies written in English | Studies written in other languages |
| Publication type | Peer-reviewed studies | Non-peer-reviewed studies |

**Table 2. Preliminary search technique**

|  | **P** | **I** | **O** | **S** |
| --- | --- | --- | --- | --- |
|  | **Population**  *Parents of adolescents* | **Interest**  *Parent-adolescent communication on sexual health and relationships* | **Outcome**  *Barriers and facilitators* | **Setting**  *United Kingdom* |
| **Boolean operators** | **AND** | **AND** | **AND** | **AND** |
| **OR** | Parents | Parent-child Relations | Communication barriers | United Kingdom |
| **OR** | Parent | Communication | Communication skills | Britain |
| **OR** | Caregiver | Sexual Health | Health Knowledge | England |
| **OR** | Care giver* | Sex Education | Attitude to sexuality | Scotland |
| **OR** | Mother | Sexually Transmitted Diseases | Barriers | Wales |
| **OR** | Father | Sexual Behaviour | Obstacles | Northern Ireland |
| **OR** | Adolescence | Pregnancy in Adolescence | Challenges |  |
| **OR** | Adolescen* | Communicat* | Difficulties |  |
| **OR** | Teen* | Talk* | Issues |  |
| **OR** | Youth | Discuss* | Problems |  |
| **OR** | Young person | Sex* | Attitudes |  |
| **OR** | Young people | STI | Facilitators |  |
| **OR** |  | Pregnancy | Enablers |  |
| **OR** |  |  | Influenc* |  |
| **OR** |  |  | Factors |  |
|  |  |  | Determinants |  |

**Table 3. Data extraction form**

| **Citation** | Full reference of article including author names, year and source. |
| --- | --- |
| **Country of study** |  |
| **Date of extraction** |  |
| **Reviewer name** |  |
| **Purpose/objectives of study** | The purpose of study is outlined by the author of the article |
| **Study design**  -Type of study  - Duration of study  - Country of origin | Type of qualitative or quantitative study used as well as the duration of study and country where the study was conducted |
| **Study population**  - Number of participants  -Type of participants  - Level of education | Description of the study sample/sample size |
| **Study outcomes** | -Barriers  -Facilitators |
| **Results of the study** | Details of the research findings |

**Table 4. The Critical Appraisal Skills Programme Qualitative (CASP) checklist**

| **Questionnaire for validity** | **Yes** | **Can’t Tell** | **No** | **Hint** |
| --- | --- | --- | --- | --- |
| 1.Was there a clear statement of the aims of the research? |  |  |  | • what is the aim, rationale and relevance for the study? |
| 2. Is a qualitative methodology appropriate? |  |  |  | • What is the right qualitative methodological approach for the study? |
| 3.Was the research design appropriate to address the aims of the research? |  |  |  | • What was the rationale for justifying the research design? |
| 4.Was the recruitment strategy appropriate to the aims of the research? |  |  |  | Is there a justified (appropriate) rationale for the participants’ selection? |
| 5.Was the data collected in a way that addressed the research issue? |  |  |  | • Is there a justification for data collection, saturation and the methods employed/chosen? |
| 6.Has the relationship between researcher and participants been adequately considered? |  |  |  | • What is the potential bias on the research question, data collection and study setting? |
| 7. Have ethical issues been  taken into consideration? |  |  |  | What are the ethical issues in the study and how were they addressed? |
| 8. Was the data analysis  sufficiently rigorous? |  |  |  | Is there clarity/bias in how the data is analysed and findings presented? |
| 9. Is there a clear statement of findings? |  |  |  | • How critical and explicit are the findings discussed? |
| 10. How valuable is the research? |  |  |  | What is the contribution of the research to existing knowledge, practice or policy? How transferable are the findings? |
